# Long noncoding RNA BX357664 regulates cell proliferation and epithelial-to-mesenchymal transition via inhibition of TGF-β1/p38/HSP27 signaling in renal cell carcinoma

**DOI:** 10.18632/oncotarget.12937

**Published:** 2016-10-27

**Authors:** Yiyang Liu, Jian Qian, Xiao Li, Wei Chen, Aiming Xu, Kai Zhao, Yibo Hua, Zhengkai Huang, Jianzhong Zhang, Chao Liang, Shifeng Su, Pu Li, Pengfei Shao, Jie Li, Chao Qin, Zengjun Wang

**Affiliations:** ^1^ Department of Urology, The First Affiliated Hospital of Nanjing Medical University, Nanjing, China

**Keywords:** lncRNA BX357664, long noncoding RNA (LncRNA), epithelial-to-mesenchymal transition, TGF-beta 1/p38/HSP27 signaling, renal cell carcinoma

## Abstract

Antisense long noncoding RNAs (lncRNAs) are reported to play a regulating role in carcinogenesis of various human malignancies. However, the function of lncRNAs and their underlying mechanism in renal cell carcinoma (RCC) is still unknown. The aims of this study are to investigate the expression of lncRNA BX357664 in RCC and to explore its function in RCC cell lines. As a result, BX357664 was downregulated in RCC according to previous microarray analysis and qualitative real-time polymerase chain reaction. After the upregulation of BX357664, reduced migration, invasion, and proliferation capabilities in RCC cells were detected. Furthermore, Western blot analysis was conducted to identify the influence of BX357664 on epithelial-to-mesenchymal transition, matrix metalloproteinase 2, matrix metalloproteinase 9, and transforming growth factor-beta 1 (TGF-β1)/p38/HSP27 signaling pathway in RCC. Subsequently, upregulating the protein level of TGF-β1 in the presence of BX357664 could rescue the suppression of the malignant behavior mediated by BX357664, indicating that BX357664 attributed its inhibitory role to the suppression of TGF-β1. Therefore, we investigated a novel lncRNA BX357664, which might exhibit its inhibitory role in RCC metastasis and progression by blocking the TGF-β1/p38/HSP27 pathway.

## INTRODUCTION

Renal cell carcinoma (RCC) accounts for 2% to 3% of all malignancies in Western countries and represents approximately 90% of all kidney cancers [1, 2]. Approximately 84,400 new cases were diagnosed with RCC and 34,700 deaths were estimated in Europe in 2012 [1]. The most effective method to cure RCC is surgical treatment because of its high resistance to conventional chemotherapy and radiotherapy [3]. However, approximately 20% to 40% of patients still develop metastasis or local recurrence after nephrectomy, with a median survival of only 6 months to 12 months and a 5 year survival of 9% [4, 5]. Thus, early diagnosis and prognostic biomarkers are essential to patients with RCC.

Only <3% of the human DNA sequence encodes protein, whereas more than 80% of human genome could exhibit biochemical functions with no protein-coding capability which defined as noncoding RNAs(ncRNAs) [6, 7]. ncRNAs are basically divided into two groups based on their size, namely, small noncoding RNAs and long noncoding RNAs (lncRNAs). Small noncoding RNAs, particularly microRNAs, have been extensively investigated for several decades, and their biological functions in various cancers have been uncovered [8]. However, the function of lncRNAs in numerous cancers still remains unknown.

lncRNAs are defined as endogenous RNAs with size larger than 200 nucleotides, but lack open reading frames of protein-coding capability [9]. lncRNAs are considered “transcription noise,” which are of no use in biological processes. Recently, a series of studies reported that lncRNAs are involved in gene regulation at different levels, such as epigenetic modification, transcription, and posttranscription [10]. Accumulating information has shown that a large number of lncRNAs exerts its tissue specificity in various cancers and plays an important role in carcinogenesis, tumor progression, and metastasis [11-17]. However, only a few studies reported the regulating role of lncRNA in RCC. Thus, analyzing the biological effects and underlying molecular mechanism of lncRNAs in RCC is significant.

Transforming growth factor-beta (TGF-β) is a member of a superfamily with more than 40 secreted cytokines [18-20]. Numerous studies have shown that TGF-β is related to carcinogenesis and tumor progression. TGF-β has been reported to function as a tumor promoter by inducing epithelial-to-mesenchymal transition (EMT), which can promote invasion and migration in various cancer cell lines [21, 22]. EMT has long been considered an essential event in morphogenesis during embryonic progression by developmental biologists [23]. Recently, a number of studies have revealed that EMT plays a key role in promoting cancer progression and metastasis. EMT features the loss of epithelial phenotypes, such as E-cadherin and claudins, and the acquisition of mesenchymal phenotypes, such as N-cadherin, vimentin, and fibronectin [21, 24, 25]. As a result, epithelial cancer cells lose their cell–cell adhesive junctions and epithelial polarity while reorganizing their cytoskeleton and acquiring mesenchymal characteristics that allow them to go through the basement membrane and generate their distant metastasis [21]. Recent studies have identified several signaling pathways for TGF-β-induced EMT [22]. In particular, the TGF-β/p38 mitogen-activated protein kinase (MAPK) signaling pathway is a well-investigated inducer of EMT in a variety of cancers, including lung cancer, renal cancer, breast cancer, and liver cancer [26-29].

In a preliminary study, we provided an overview of the differential expression patterns of lncRNAs by lncRNA microarray in five RCC patients [30]. From the analysis of normalized microarray data and further qualitative polymerase chain reaction (qPCR) identification, we determined that lncRNA BX357664 (see the details in the “Discussion” section) might be a potential biomarker involved in RCC. Therefore, we conducted further experiments on RCC cell lines to investigate the role of BX357664 in RCC.

We used qPCR to identify the levels of BX357664 in RCC samples and RCC cell lines. Then, we investigated the role of BX357664 in tumor metastasis and proliferation by in vitro assays. We evaluated the hallmarks of EMT and proteins in the TGF-β/p38 MAPK/HSP27 signaling pathway to determine how BX357664 affects cell migration and invasion.

The results of this study may reveal how BX357664 acts as a tumor suppressor in RCC cell lines and may provide a potential biomarker for the diagnosis and treatment of RCC.

## RESULTS

### BX357664 is tissue-specific and downregulated in RCC tissues and cells

**Figure 1:**
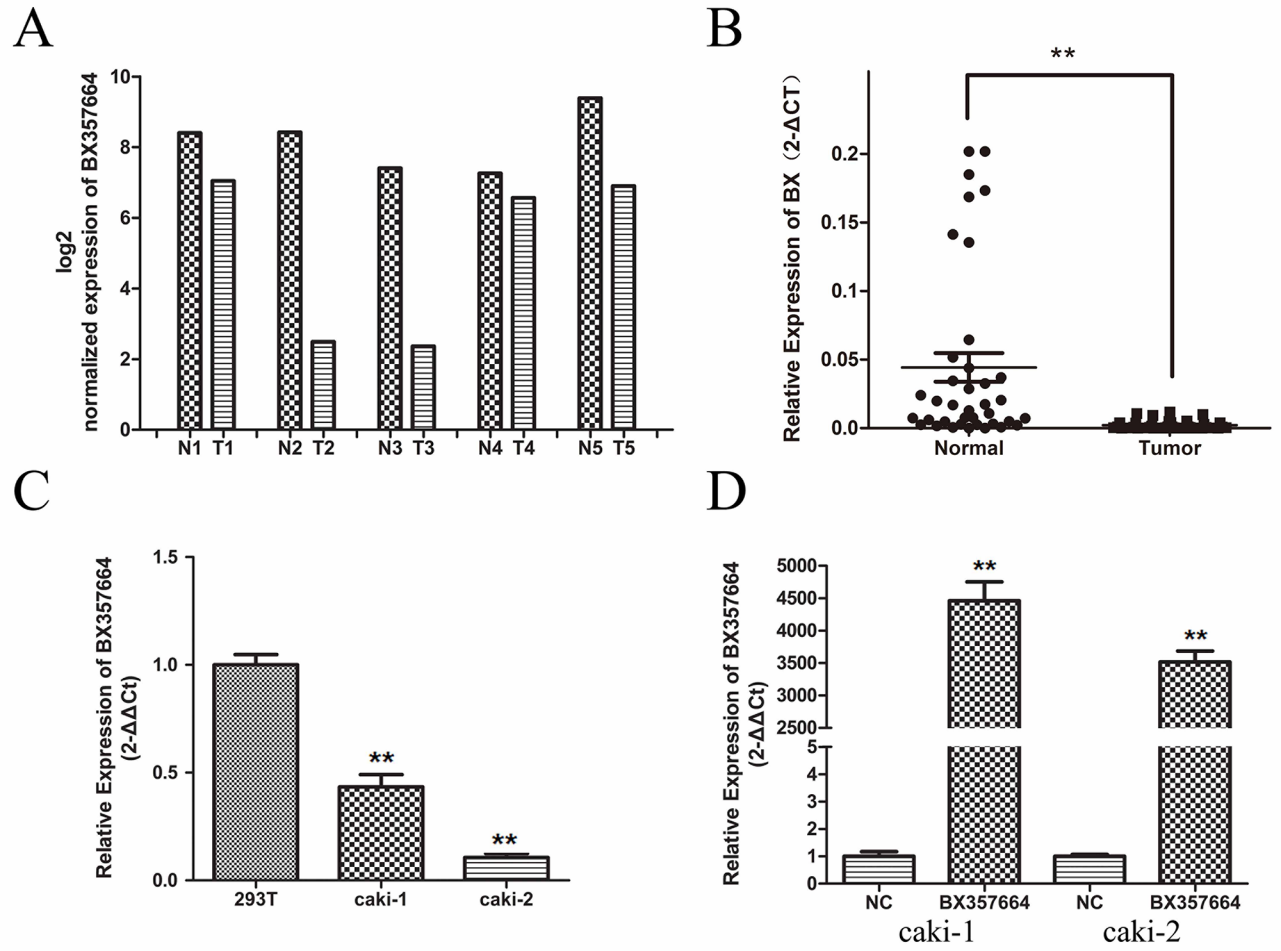
BX357664 is downregulated in RCC BX357664 level in RCC samples was significantly downregulated compared with the paired adjacent normal tissues according to microarray data **A.** and qRT-PCR **B. C.** The expression level of BX357664 in RCC cell lines was relatively low compared with the normal renal cell line. **D.** Relative expression of BX357664 in transfected cells. The expression of BX357664 was upregulated in the transfected Caki-1 and Caki-2 cells. The median in each triplicate was used to calculate the BX357664 concentration using either the comparative 2^−ΔΔCt^ or 2^−ΔCt^ method. *p < 0.05 and **p < 0.01 compared with the adjacent normal tissues.

According to the standardized analysis of lncRNA microarray data, the expression level of BX357664 in tumor tissues was remarkably lower than that in adjacent normal tissues (p < 0.05; Figure 1A).

Qualitative real-time polymerase chain reaction (qRT-PCR) was conducted to investigate BX357664 expression in 38 paired RCC tissues and adjacent normal tissues to validate the deregulation of BX357664 from microarray data(p < 0.01; Figure 1B). TaqMan RT-PCR method was also conducted to confirm this expression pattern. The result was consistent with the microarray data(p < 0.01; Supplementary Figure S1).

In addition, the expression level of BX357664 was also determined in two RCC cell lines (Caki-1 and Caki-2) and in the normal renal cell line (HEK-293T). The expression of BX357664 was significantly lower in Caki-1 and Caki-2 than that in HEK-293T, corresponding to the result in tissues (p < 0.01; Figure 1C).

**Figure 2:**
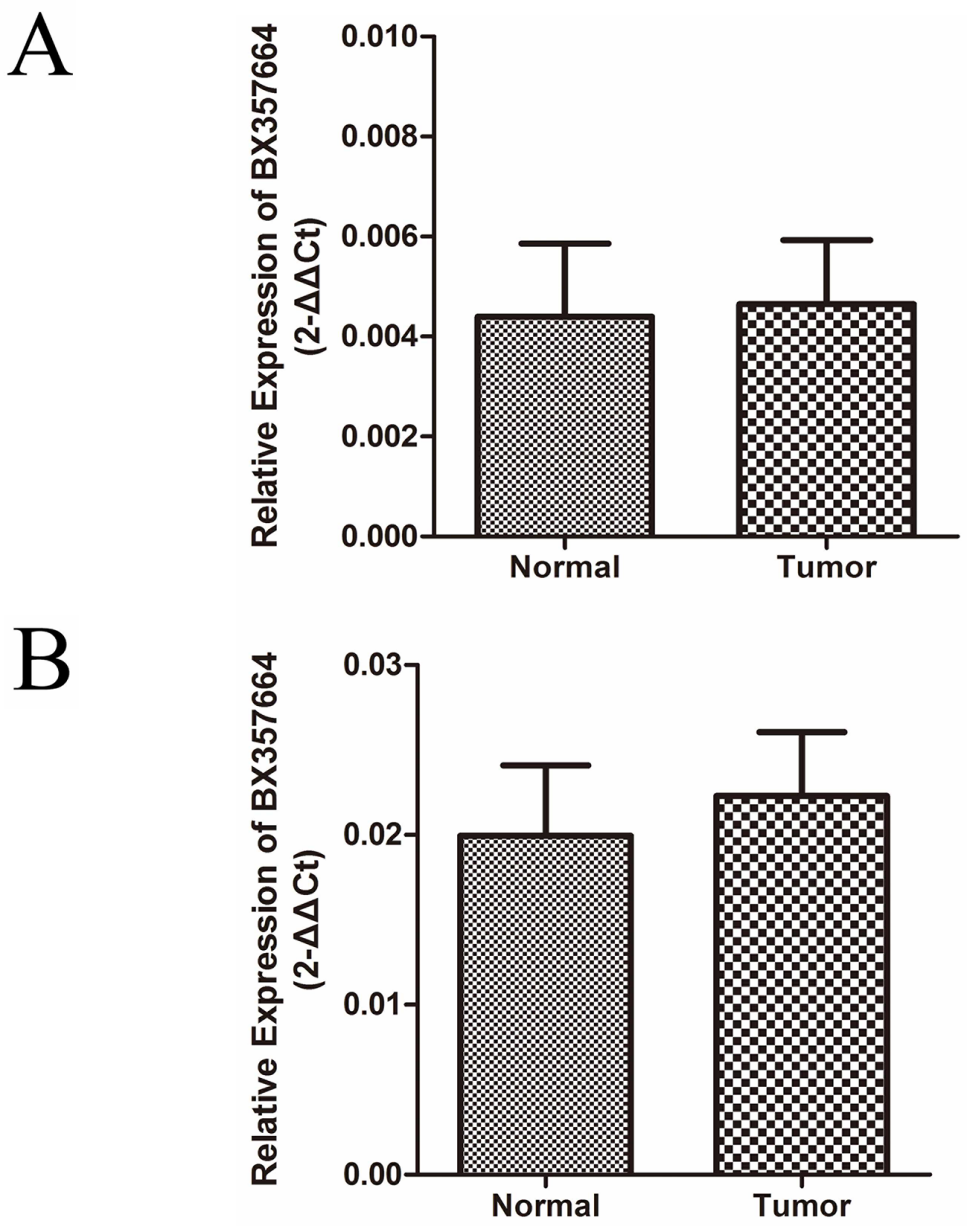
BX357664 is not differentially expressed in bladder tumor and prostate cancer The expression of BX357664 showed no difference between tumor tissues and normal tissues in bladder tumor **A.** and prostate cancer **B.** The median in each triplicate was used to calculate the BX357664 concentration using the comparative 2^−ΔCt^ method. *p < 0.05 and **p < 0.01 compared with the adjacent normal tissues.

qRT-PCR was further determined to detect the expression pattern of BX357664 in 8 paired bladder tumor tissues, prostate cancer tissues and adjacent normal tissues. The expression of BX357664 showed no statistical difference between tumor tissues and normal tissues, indicating that BX357664 might exert its tissue specific phenotype in RCC, rather than bladder tumor and prostate cancer in urologic malignancies(p > 0.05; Figure 2).

### Inhibition of BX357664 on cell proliferation

**Figure 3:**
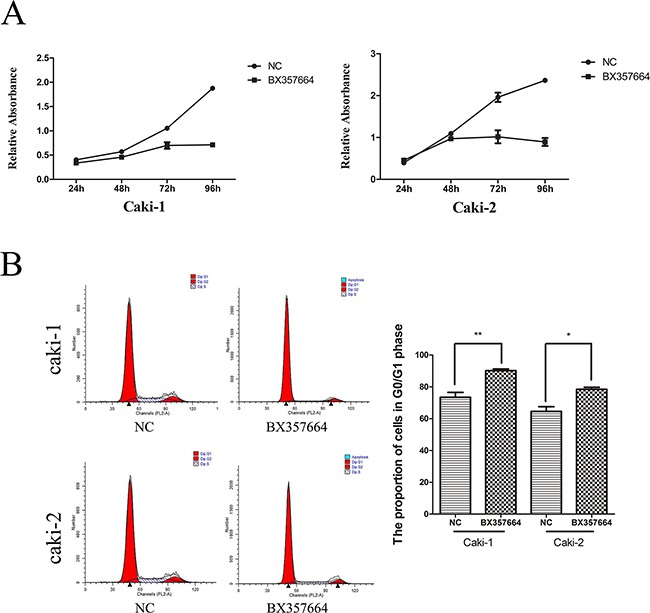
BX357664 inhibits cell proliferation and induces cell cycle arrest in the Caki-1 and Caki-2 cell lines **A.** Cell proliferation by CCK-8 assay. The proliferation of Caki-1 and Caki-2 cells was significantly inhibited by the upregulation of BX357664. **B.** Flow cytometry analysis showed cell cycle arrest of Caki-1 and Caki-2 cells at the G1 phase after the overexpression of BX357664. The histogram indicates the percentage of cells in G1. *p < 0.05 and **p < 0.01 compared with the negative control group.

CCK8 assay indicated that the growth of Caki-1 and Caki-2 cells were significantly inhibited after the upregulation of BX357664, compared with the NC group (p < 0.05; Figure 3A).

In addition, flow cytometry analysis showed that the percentages of Caki-1 and Caki-2 cells transfected with BX357664 lentivirus in the G0/G1 phase were remarkably higher than that transfected with NC lentivirus, indicating that BX357664 could promote G0/G1 cell cycle arrest in RCC cells (p < 0.05; Figure 3B).

### Inhibition of BX357664 on cell migration and invasion

**Figure 4:**
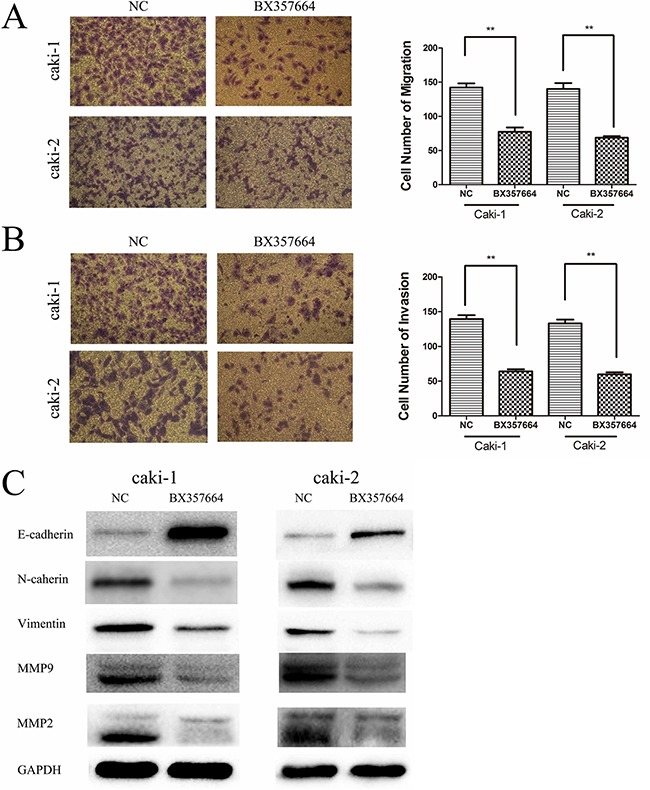
BX357664 inhibits cell migration, invasion and EMT in RCC cells **A.** and **B.** Overexpression of BX357664 inhibited migration and invasion in the RCC cell lines. **C.** BX357664 blocks EMT, MMP2 and MMP9 in RCC cells. Western blot analysis was used to detect the changes in EMT markers in NC group and BX group cells. Gain in E-cadherin expression and loss of N-cadherin, vimentin, MMP2 and MMP9 were observed in BX group cells. Data are expressed as the mean ± SD of at least three independent experiments. *p < 0.05 and **p < 0.01 compared with the negative control group. Original magnification, ×200.

Migration and invasion assays were conducted to explore whether BX357664 affects the migration and invasion capabilities in Caki-1 and Caki-2. The migration assay indicated that overexpression of BX357664 could significantly suppress the migration capability in Caki-1 and Caki-2 compared with the NC group (p < 0.01; Figure 4A).

Meanwhile, the invasion assay showed that overexpression of BX357664 could inhibit the invasion capability in Caki-1 and Caki-2 compared with the NC group (p < 0.01; Figure 4B).

### Suppression of BX357664 on EMT, MMP2 and MMP9

Considering that the inhibitory role of BX357664 in RCC cell migration and invasion had been verified and EMT was previously reported to be linked with cancer metastasis, we further investigated whether BX357664 could affect EMT markers in RCC cells. Compared with the NC group, overexpression of BX357664 could remarkably influence the expression of EMT markers in the protein level, leading to the upregulation of E-cadherin and the downregulation of N-cadherin and vimentin, which indicated that BX357664 could inhibit EMT in RCC cells (p < 0.05; Figure 4C). Moreover, we also determined that BX357664 could suppress the expression of MMP2 and MMP9 in RCC cells (p < 0.05; Figure 4C), providing more evidence for the inhibitory role of BX357664 in cancer metastasis in RCC.

### Inactivation of BX357664 on TGF-β1/p38/HSP27 signaling

**Figure 5:**
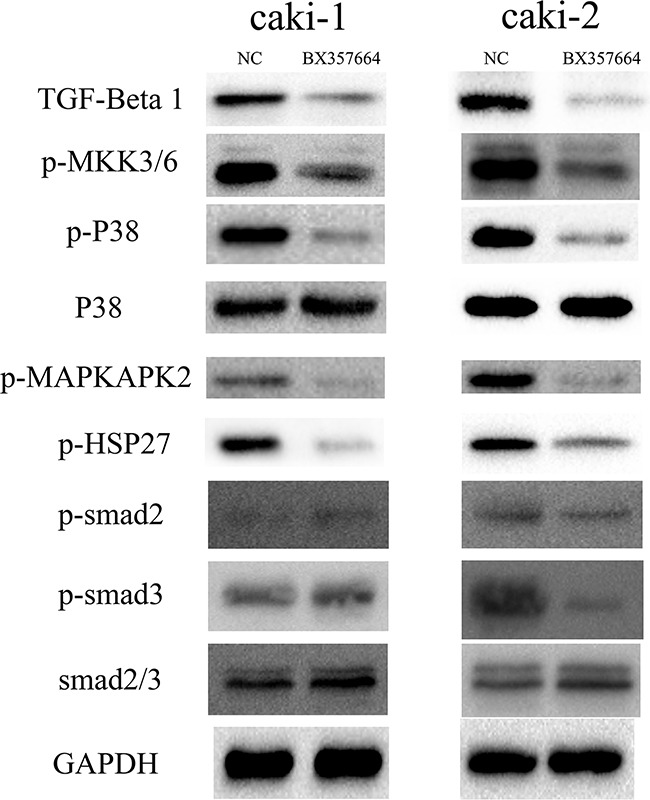
BX357664 suppresses the TGF-β1/p38/HSP27 pathway Western blot analysis of the TGF-β1/p38/HSP27 and TGF-β1/Smad pathways. In the Caki-1 and Caki-2 cells, TGF-β1, p-MKK3/6, p-p38, p-MAPKAPK2, and p-HSP27 decreased with the high expression of BX357664, whereas decreased expression of p-smad3 was only observed in Caki-2 BX group cells. *p < 0.05 compared with the negative control group.

We assessed the effect of the upregulation of BX357664 on the expression of TGF-β1, p38, and p-smad2/3 in the protein level to explore the mechanism of BX357664 in regulating EMT. TGF-β1 and p-p38 significantly decreased with the overexpression of BX357664. To the smad2/3 signaling, however, only p-smad3 was founded to be inhibited in caki-2 cell line, rather than caki-1, which might due to cell-specific difference. Therefore, it indicated that BX357664 might suppress RCC metastasis mainly through TGF-β1/p38 pathway, rather than smad-dependent TGF-β pathway. A series of effectors in this pathway were evaluated to confirm the inactivation of TGF-β1/p38 signaling induced by BX357664 further. The result showed that p-MKK3/6, p-MAPKAPK2, and p-HSP27 significantly decreased with the upregulation of BX357664, indicating that BX357664 indeed inactivated TGF-β1/p38/HSP27 signaling by inhibiting the phosphorylation of effectors in this pathway (p < 0.05; Figure 5).

### Rescue experiment on cell proliferation, migration, and invasion

After the transfection of BX357664, cells were stimulated with recombinant human transforming growth factor-beta 1 (Rh TGF-β1) to upregulate the protein level of TGF-β1 to verify if tumor behavior could be rescued by restoring TGF-β1 expression in the presence of BX357664 overexpression.

**Figure 6:**
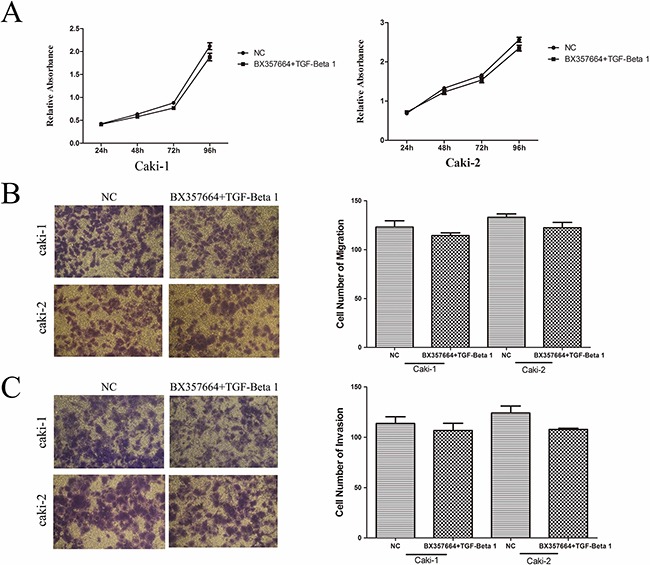
Observation of cell proliferation, migration and invasion after Rh TGF-β1 stimulation in BX group cells **A.** Cell proliferation by CCK-8 assay. The inhibitory role of BX357664 on the proliferation of Caki-1 and Caki-2 cells was attenuated after Rh TGF-β1 stimulation. **B.** and **C.** The inhibitory role of BX357664 on the migration and invasion of Caki-1 and Caki-2 cells was attenuated after Rh TGF-β1 stimulation. p > 0.05 compared with the negative control group.

CCK8 assay demonstrated that the inhibition of cell proliferation caused by BX357664 was rescued by stimulation with Rh TGF-β1. No statistically significant difference was observed between BX357664+ Rh TGF-β1 group and NC group in Caki-1 and Caki-2 cell lines (p > 0.05; Figure 6A).

In migration and invasion assays, decreased migration and invasion capabilities induced by BX357664 were also alleviated after Rh TGF-β1 stimulation. As shown in Figure 6, no significant difference was observed between BX357664+ Rh TGF-β1 group and NC group in Caki-1 and Caki-2 cell lines (p > 0.05; Figure 6B; 6C).

**Figure 7:**
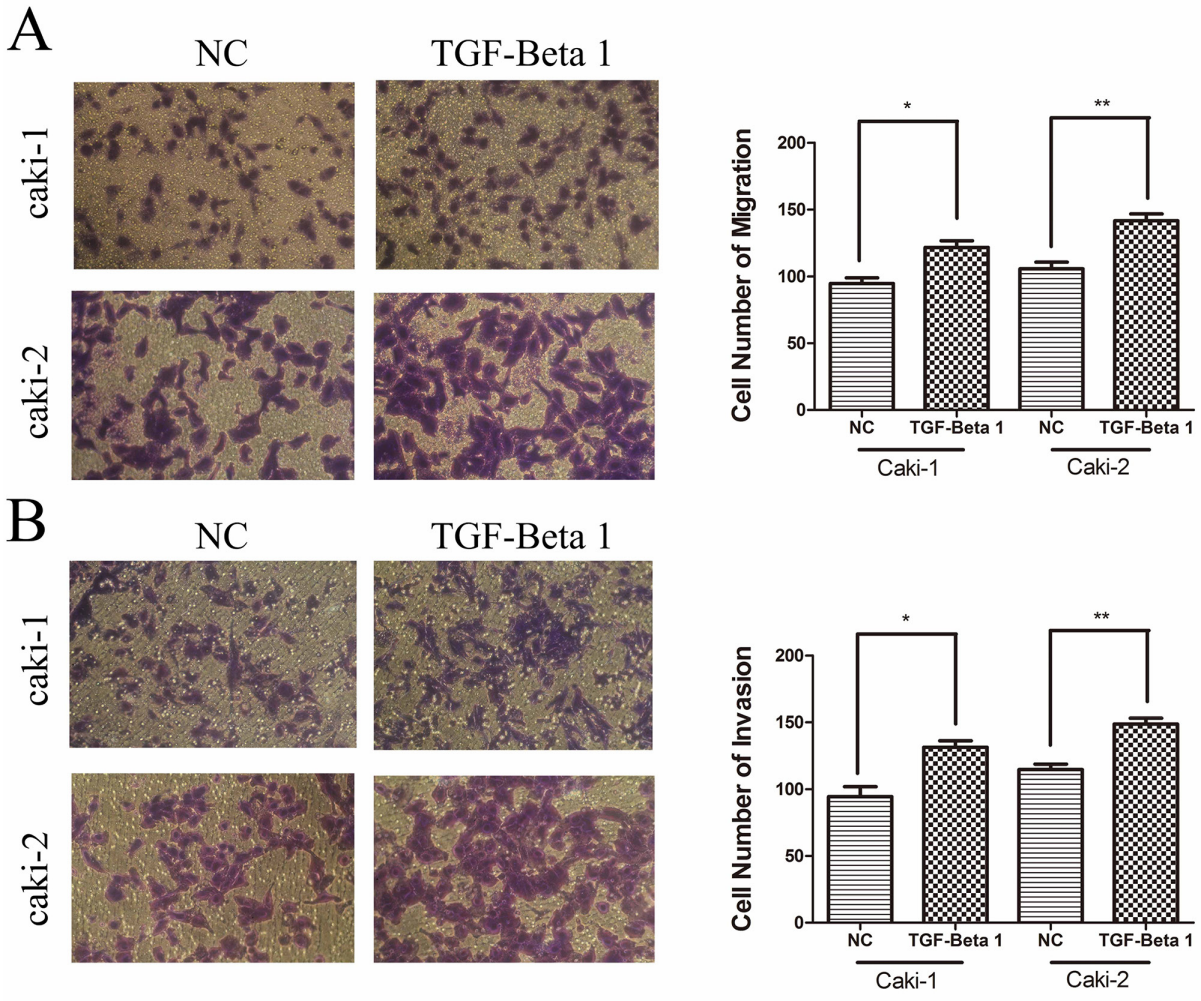
TGF-β1 promotes cell migration and invasion in RCC cells **A.** and **B.** TGF-β1 promotes migration and invasion in the RCC cell lines. Data are expressed as the mean ± SD of at least three independent experiments. *p < 0.05 and **p < 0.01 compared with the negative control group. Original magnification, ×200.

Furthermore, rhTGF-β1 was used to stimulate caki-1 and caki-2 cells respectively. Migration and invasion assays were determined to show the promotion effect of TGF-beta 1 on migration and invasion (p < 0.05; Figure 7).

These results illustrated that the inhibitory role of BX357664 in RCC might function by attenuating the expression of TGF-β1.

## DISCUSSION

Recently, the regulating role of lncRNAs in cancer genesis and progression has been gradually unraveled and investigated. According to current research reporting on the relationship between lncRNAs and urologic malignant tumor, we could divide these lncRNAs into three subgroups, namely, (1) tumor promoter; (2) tumor suppressor; and (3) tumor promoter/suppressor. As a tumor promoter, MALAT-1 could be transcriptionally activated by c-Fos and could interact with Ezh2, silencing the tumor suppressor gene E-cadherin and upregulating β-catenin expression that induce tumor promotion in RCC [31]. As a tumor suppressor, MDC1-AS inhibited the migration, invasion, and proliferation capabilities of bladder cancer cells by upregulating its antisense tumor-suppressing gene MDC1 [32]. As a tumor promoter/suppressor, we determined that, in different types of tumors, some lncRNAs could exert completely opposite regulating roles. For instance, H19 was upregulated in bladder cancer and could promote cancer metastasis [16]. Meanwhile, H19 could serve as precursor of the tumor suppressor gene microRNA-675, attenuating cancer cell motility through the downregulation of TGF-β1 in prostate cancer [17]. Low expression of H19 was also reported in Wilm's tumor [33].

**Figure 8:**
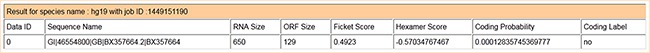
Analysis of the Coding Potential Assessment Tool revealed that BX357664 has no protein-coding probability

In a previous study, microarray analysis of lncRNA expression revealed a host of differentially expressed lncRNAs between RCC tissues and adjacent normal tissues [30]. Initially, we focused on lncRNA CR613822, which was downregulated in RCC according to the data. However, the information on lncRNA CR613822 was deleted by the uploader of the NCBI nucleotide database. Meanwhile, the updated details referred to the BX357664 gene. The coding probability of BX357664 was predicted online using the Coding Potential Assessment Tool, indicating that BX357664 did not have the potential of coding protein [34] (Figure 8). Moreover, the length of the sequence of BX357664 is 650 nt. Therefore, we assumed that BX357664 is a lncRNA according to the definition.

Considering the fact that BX357664 is downregulated in RCC tissues detected by microarray analysis, we further investigated the BX357664 levels in 38 paired RCC tissues and cell lines. We found that the expression of BX357664 was significantly downregulated in RCC. Moreover, we found no deregulated expression pattern of BX357664 in bladder tumor and prostate cancer. It indicated that BX357664 might be a newly identified differentially expressed lncRNA in RCC and possess tissue specificity, which might exert its function in regulating the process of RCC.

At present, only a few studies reported that lncRNA functioned as a tumor suppressor in RCC. GAS5 could induce apoptosis, inhibit cell proliferation and metastasis in A498 cells. These conclusions provided convincing evidence for lncRNA serving as a tumor suppressor in RCC [14]. Thus, we assumed that the upregulation of BX357664 in RCC cell lines could also have an antitumor effect.

At this point, we transfected BX357664 lentivirus and negative control lentivirus into RCC cell lines. We detected that BX357664 significantly inhibited cell proliferation, caused by arrest in G1 phase of the cell cycle. A previous study reported that aberrant induction of EMT in cancer cells is related to malignant tumor behavior, such as enhanced migration, invasion, and metastasis capabilities [35]. MMP2 and MMP9 are members of the matrix metalloproteinase family that can degrade the basement membrane, inducing cell invasion [36]. They are key elements involved in tumor metastasis and are linked with poor prognosis in RCC [37]. We found that the overexpression of BX357664 could suppress the migration and invasion in RCC cells by blocking the process of EMT, downregulating the expression of MMP2 and MMP9. These results verified our assumption that BX357664 might function as a tumor suppressor in RCC.

A growing body of evidence supports the finding that TGF-β1 plays a significant role in inducing EMT through various signaling pathways [21-25]. Our result revealed that TGF-β1 is downregulated in the protein level with the upregulation of BX357664. However, BX357664 did not affect TGF-β1 in the mRNA level, indicating that BX357664 might downregulate TGF-β1 through posttranscriptional regulation, other than transcriptional regulation(p > 0.05; Supplementary Figure S2).

TGF-β1/MAPK signal transduction pathways have been recently implicated to play key roles in carcinogenesis and cancer metastasis [38]. p38 MAPK is one of these pathways that can be activated through phosphorylation in response to environmental stress, inflammation, and TGF-β signaling [39]. A number of studies have proposed that the activation of p38 MAPK contributes to EMT and the overexpression of MMP9 in many tumors, offering cancer cells enhanced invasion and migration capabilities [40-44]. MKK3 and MKK6 are located upstream of p38 and are identified as essential MAPKKs that can phosphorylate and activate p38 MAPK [45]. As a downstream effector of p38, MAPKAPK2 can be activated by phosphorylation of p38, which, in turn, phosphorylates HSP27 [46]. HSP27, a member of the heat shock protein family, plays a fundamental role in various signaling pathways linked with carcinogenesis, metastasis, drug resistance, and clinical outcomes of many cancers. Deregulated phosphorylation of HSP27 and overexpression of HSP27 have been determined to be associated with cancer development, particularly in the mediation of cancer metastesis as well as EMT [47-50]. Consequently, TGF-β1/MKK3/6/p38/MAPKAPK2/HSP27 forms a signaling axis involved in cancer promotion. In this study, we identified that the overexpression of BX357664 could lead to the inactivation of the TGF-β1/p38 MAPK pathway, eventually attenuating the invasion and migration capabilities and EMT in RCC.

In rescue experiment, we found that TGF-β1 could contribute to the progression of migration and invasion in RCC and the inhibitory role of BX357664 on tumor behavior could be attenuated by TGF-β1, which indicated that BX357664 might exert its antitumor function through TGF-β1/p38 signaling in a non-smad-dependent manner.

Interestingly, we have also tested the role of BX357664 in the other two RCC cell lines, 786-O and ACHN. In 786-O cells, cck8 assay and transwell assay showed no significant difference between NC group and BX357664 group(p > 0.05; Supplementary Figure S3A and S3B). However, in Western Blot assay, BX357664 could inhibited the EMT process by inhibiting EMT markers and downregulated TGF-Beta 1 and p-p38 (Supplementary Figure S3C). In ACHN cells, cck8 assay showed BX357664 could significantly inhibit cell proliferation in time-point of 96h (Supplementary Figure S4). These results could add evidence that BX357664 was indeed a tumor suppressor in RCC.

However, our study has several limitations. Although we have detected the downregulated expression of TGF-β1 induced by BX357664 in the protein level rather than the mRNA level, the specific mechanism underlying this posttranscriptional regulation still remains unsolved. In vivo assays were required to confirm the inhibitory role of BX357664 in RCC further.

In summary, our results indicated that BX357664 is significantly downregulated in RCC and may serve as a potential tumor suppressor in RCC by inhibiting the TGF-β1/p38/HSP27 signaling pathway and by interfering with cancer invasion and metastasis in RCC. In addition, BX357664 could attenuate cell proliferation and induce cell cycle arrest in the G0/G1 phase. Consequently, BX357664 might serve as a competent candidate for molecular target therapy in RCC. Further research should be conducted in the future.

## MATERIALS AND METHODS

### Cell culture and tissue samples

The human RCC cell line (caki-1), normal human emborynic kidney cells (HEK-293T) were purchased from the Cell Bank Type Culture Collection of the Chinese Academy of Sciences (Shanghai, China), human RCC cell line(caki-2) was purchased from the China Infrastructure of Cell Line Resources. Cells of the caki-1 and caki-2 were maintained in McCoy's 5A (Gibco, USA) and cells of HEK-293T were maintained in Dulbecco's modified Eagle's medium (Gibco, USA), all supplemented with 10% fetal bovine serum (FBS, Gibco, USA) within a humidified atmosphere containing 5% CO2 at 37 °C.

Following the Local Ethics Committees of the First Affiliated Hospital with Nanjing Medical University, China, 38 paired tumor specimens and tissue samples used to assess BX357664 expression were obtained with informed consent from RCC patients (Table 1). All the patients had undergone radical nephrectomy or partial nephrectomy. All samples were obtained during surgery, immediately frozen in liquid nitrogen, and stored at –80 °C for further analysis. The identification of tumor tissues and adjacent normal tissues were confirmed by the pathologists.

**Table 1:** Characteristics of RCC patients

Gender	Male	28
	Female	10
Age, median (range)		58.5 (29-80) y
T stage	T1	31
	T2	2
	T3	5
	T4	0
Pathology	Clear cell RCC	38

### Cell transfection

The lentiviral vector with overexpression of BX357664 was constructed by Genechem (Shanghai, China). The lentiviral vector alone was used as a negative control for transfection.

Cells of the caki-1 and caki-2 were seeded in 6-well plates at 40% confluence on the day before transfection. The lentiviral vector constructs with overexpression of BX357664 and lentiviral vector alone were used to infect caki-1 and caki-2 cells at a multiplicity of infection (MOI) of 10. Three days after infection, GFP expression was detected to assess the infection efficiency. Five days after infection, cells were harvested into two parts. Real-time reverse transcription polymerase chain reaction (RT-PCR) was performed to evaluate BX357664 expression efficiency in one part of cells, while the other part was used for future cell amplification and experiments. Cells overexpressing BX357664 were defined as BX group, while cells transfected with lentiviral vector alone were defined as NC group.

### Recombinant human TGF-beta 1 stimulation

Recombinant Human TGF-beta 1(Rh TGF-beta 1) (R&D system, USA) was reconstituted at 20 μg/mL in sterile 4 mM HCl containing 0.1% bovine serum albumin for storing and use. Cells of the BX group cells were seeded in 6-well plates at 50% confluence on the day before stimulation. 48 hours after Rh TGF-beta 1 stimulation at concentration of 2ng/mL, cells were harvested for further experiments. BX group cells stimulated with Rh TGF-beta 1 were defined as BX+TGF-Beta 1 group.

### RNA isolation and qRT-PCR

Total RNA was isolated from tissues and cultured cells using Trizol (Invitrogen, USA) in accordance with the manufacturer's instructions for lncRNA and mRNA analyses. RNA concentration was measured using NanoDrop (Thermo Scientific, USA).

cDNA was synthesized from total RNA using High capacity RNA-to-cDNA kit (Applied Biosystems, USA) according to the manufacturer's protocol. Analysis of BX357664 expression was performed by qRT-PCR using a SYBR Green assay in accordance with the manufacturer's instructions (Applied Biosystems, USA). Relative expression of BX357664 were calculated using the 2^-ΔΔCt^ method. The primers were designed as follows: BX357664, 5’-GGCGTGGTTTTGATGGAGTG-3’, 5’-A GGCTGCAGAGTTGAGATCG-3’; β-actin, 5’-ACTGGA ACGGTGAAGGTGAC-3’, 5’-AGAGAAGTGGGGTG GCTTTT-3’. qRT-PCR was performed under the following conditions: 50 °C for 2 min, 95 °C for 2 min; 40 cycles at 95 °C for 15 s, and 60 °C for 1 min; 95 °C for 15s, 60 °C for 1 min, 95 °C for 15s. The reactions were performed and analyzed by Applied Biosystems StepOne Plus Real-Time PCR System (Applied Biosystems, USA). All reactions were run in 3 times.

### Cell proliferation assay

caki-1 and caki-2 transfected with BX357664 or NC were performed cell proliferation assay to investigate the effect of BX357664 on cell proliferative capacity. Different pretreated cells were seeded into 96-well plates at a density of 1.5×10^3^ cells/well and cultured for 24, 48, 72,and 96 h. Cell proliferation was calculated using a Cell Counting Kit-8 (CCK-8; Dojindo Molecular Technologies, Japan) in accordance with the manufacturer's protocol. Absorbance was detected at the wavelength of 450 nm. Three wells were measured for cell viability in each group.

### Cell cycle assay

Flow cytometry was adopted to analyze the distribution of cell cycle stages in transfected cells to study the potential mechanism of BX357664 on cell growth. Different pretreated cells were harvested, washed twice with phosphate buffered saline, and fixed with 70% ethanol at –20 °C overnight. Then, cells were incubated in 50 mg/mL propidium iodide and 1 mg/mL RNase for 30 min at room temperature. Lastly, treated cells were analyzed by flow cytometry (Becton Dickinson). At least 100,000 cells were acquired for each sample. The experiments were performed in triplicate.

### Cell migration and invasion assays

Migration and invasion assays were conducted to determine the function of BX357664 in RCC metastasis. For the migration assays, 2 × 10^4^ cells in 200 μL of serum-free medium were placed in the top chamber of the transwell (pore size, 8 mm; BD Biosciences, San Jose, CA, USA). For the invasion assays, 6 × 10^4^ cells in 200 μL of serum-free medium were placed in the top chamber adhered with Matrigel (BD Biosciences, San Jose, CA, USA) following the manufacturer's protocol. Then, 500 μL of media containing 20% FBS were added to the bottom chamber. After 24 h of incubation at 37 °C, the cells remaining on the top membrane were removed and those on the bottom surface of the membrane were fixed in 95% ethanol and stained with crystal violet. Five fields were randomly counted. Experiments were repeated three times.

### Protein isolation and western blot

To understand how BX357664 regulated the RCC cells metastasis and growth, we use Western blot assay to explore the potential change of signaling pathways and EMT caused by BX357664. To extract protein from transfected cells, cells were washed twice in phosphate buffered saline and lysed using radioimmunoprecipitation assay buffer (KeyGene Biotech) supplemented with protease inhibitors at 4 °C for 30 min. Same amounts quantity of proteins were analyzed by 10% sodium dodecyl sulfate polyacrylamide gel electrophoresis, transferred to a polyvinylidene fluoride membrane (Millipore, USA), blocked for 1.5 h with 5% nonfat milk at room temperature, and incubated with primary antibodies at 4 °C overnight. The membrane was incubated with a horseradish peroxidase-conjugated secondary antibody for 2 h after three washes with Tris-buffered saline and 0.1% Tween. Antibodies against GAPDH (Bioworld Technology, USA), TGF-Beta 1, p38, p-p38, MMP9, MMP2, p-MKK3/6, p-MAPKAPK2, p-HSP27, E-cadherin, N-cadherin, and vimentin (Cell Signaling Technology, USA) were used in Western blot analysis according to the manufacturer's instructions. Protein levels were calculated based on GAPDH equivalence.

### Statistical analyses

Differences between two groups were analyzed using Student's t-test. All of the statistical results were performed by SPSS (SPSS for Version 13.0). P < 0.05 was considered statistically significant.

## SUPPLEMENTARY MATERIALS FIGURES


