# Supplementary material for: Long noncoding RNA BX357664 regulates cell proliferation and epithelial-to-mesenchymal transition via inhibition of TGF-β1/p38/HSP27 signaling in renal cell carcinoma
Source: Oncotarget. 2016 Oct 27;7(49):81410–22. doi: 10.18632/oncotarget.12937 (PMC5348402; doi:10.18632/oncotarget.12937)
Supplement: Supplementary file 1 [file oncotarget-07-81410-s001.pdf]

Long noncoding RNA BX357664 regulates cell proliferation and epithelial-to-mesenchymal transition via inhibition of TGF-β1/p38/HSP27 signaling in renal cell carcinoma

SUPPLEMENTARY FIGURES

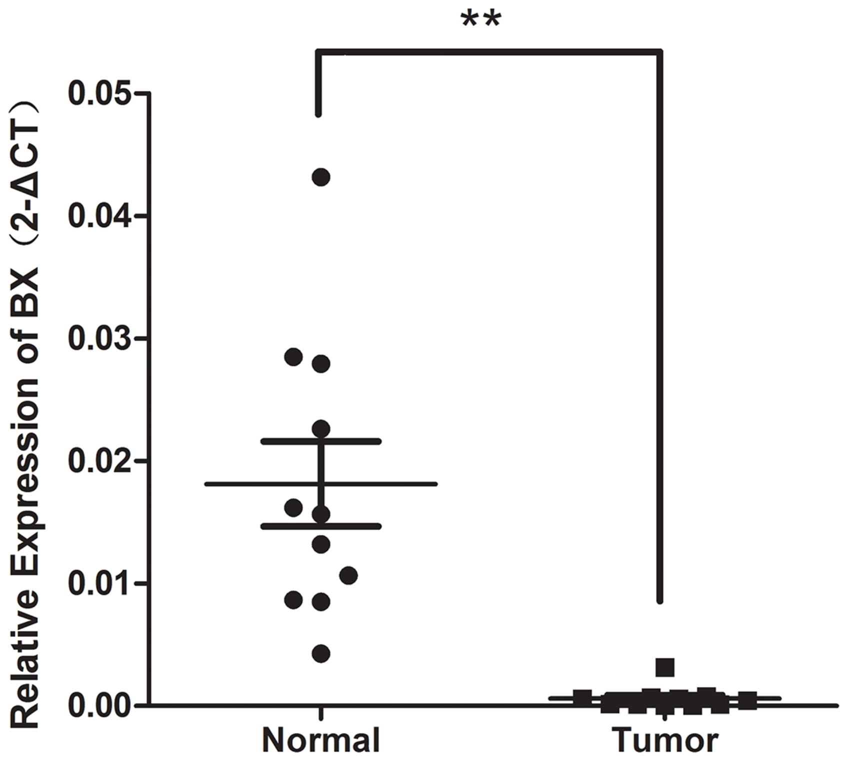

Supplementary Figure S1: BX357664 is downregulated in RCC. BX357664 level in 11 RCC samples was significantly downregulated compared with the paired adjacent normal tissues according to TaqMan RT-PCR. The median in each triplicate was used to calculate the BX357664 concentration using the comparative $2^{-\Delta Ct}$ method. *p < 0.05 and **p < 0.01 compared with the adjacent normal tissues.

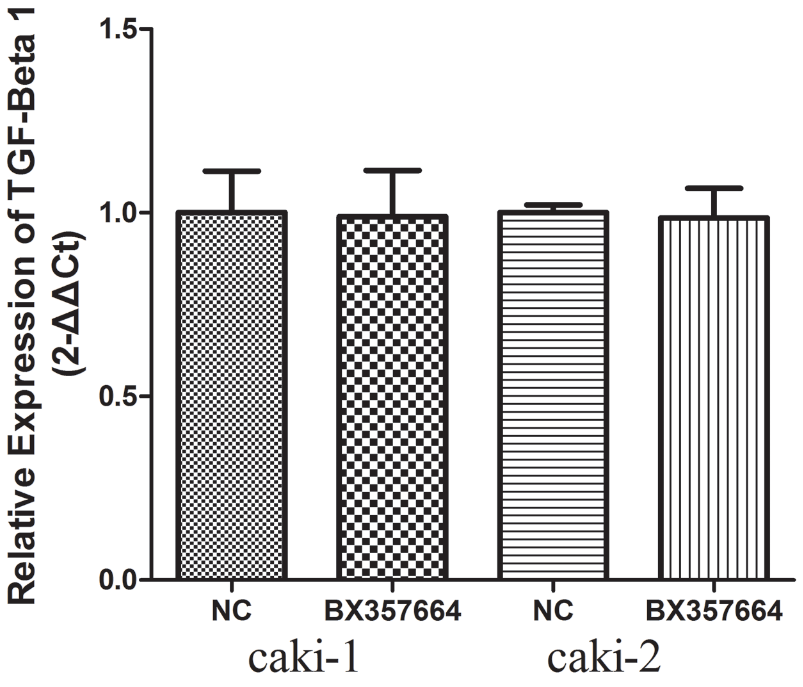

Supplementary Figure S2: TGF-β1 mRNA level is not affected by overexpression of BX357664 in RCC cells. The median in each triplicate was used to calculate the BX357664 concentration using the comparative $2^{-\Delta\Delta Ct}$ method. *$p < 0.05$ and **$p < 0.01$ compared with the adjacent normal tissues.

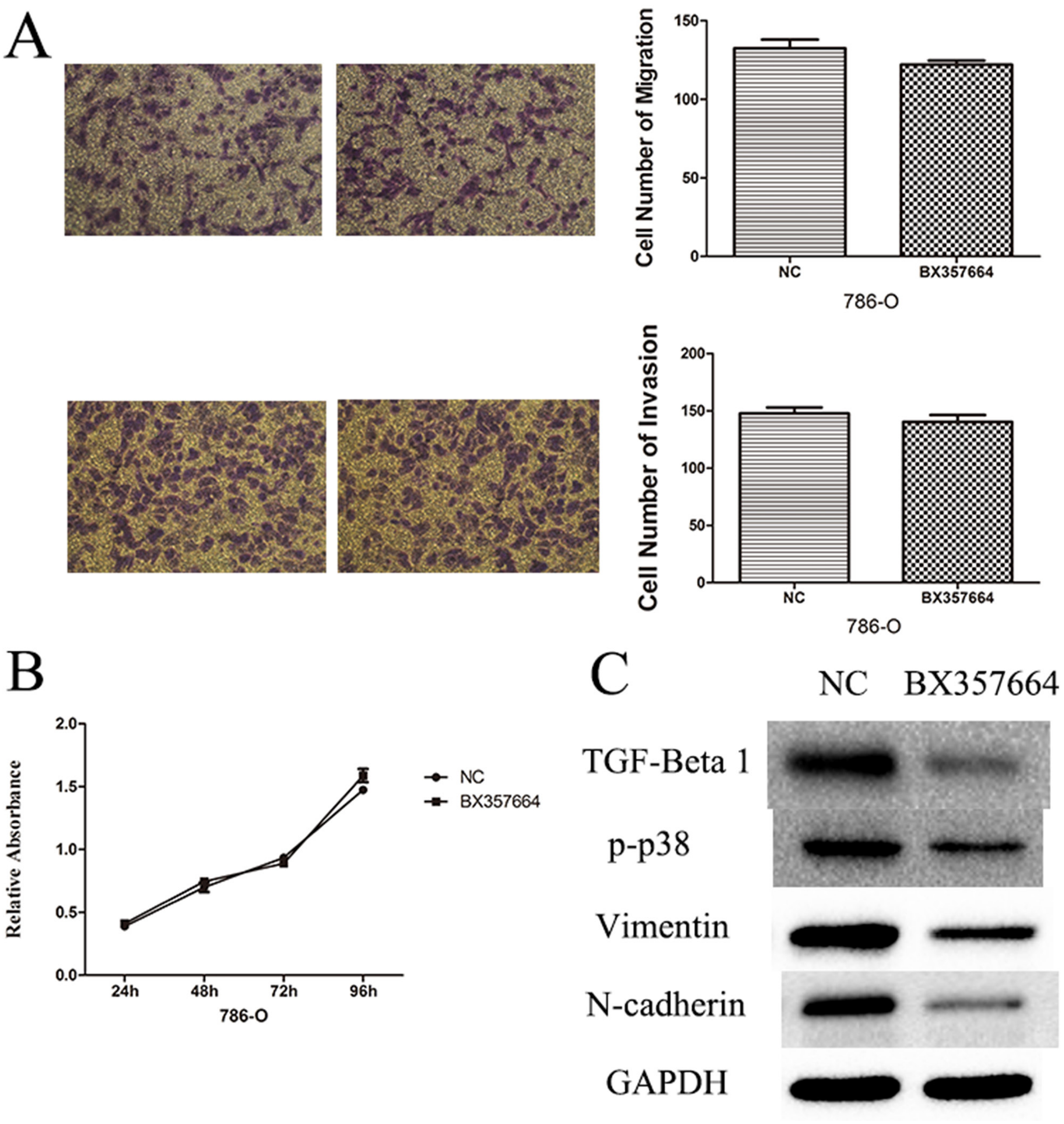

Supplementary Figure S3: BX357664 inhibits EMT while not affecting cell proliferation, migration and invasion in the 786-O cells. **A.** and **B.** BX357664 could not inhibit cell migration, invasion and proliferation in the 786-O cells. $p > 0.05$ compared with the negative control group. **C.** BX357664 blocks EMT, downregulates the expression of TGF-β1 and p-p38 in 786-O cells using Western blot analysis. $^*p < 0.05$ compared with the negative control group.

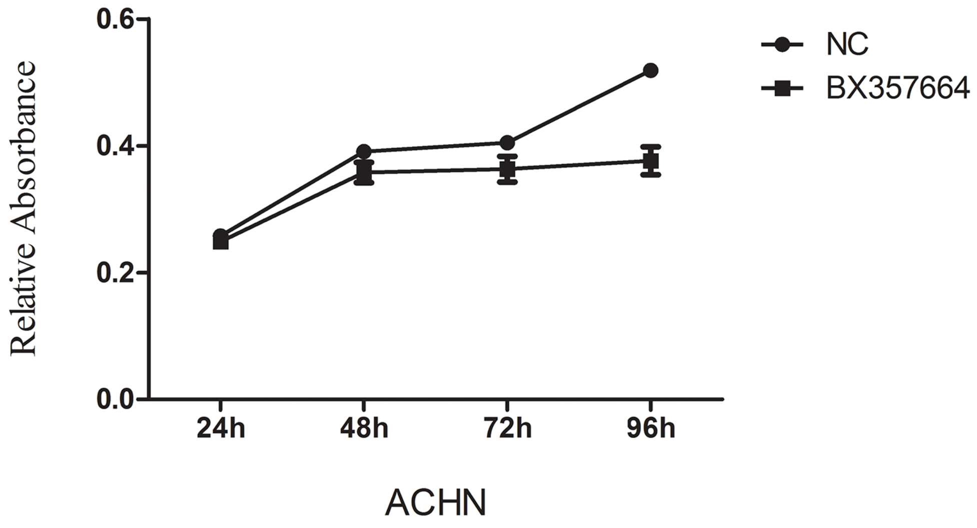

Supplementary Figure S4: BX357664 inhibits cell proliferation in the ACHN cells. Cell proliferation by CCK-8 assay. The proliferation of ACHN cells was significantly inhibited at time-point of 96h after the upregulation of BX357664. *$p < 0.05$ compared with the negative control group.
